# Diet, Sleep and Exercise: The Keystones of Healthy Lifestyle for Medical Students

**DOI:** 10.31729/jnma.7355

**Published:** 2022-09-30

**Authors:** Mamata Yadav

**Affiliations:** 1 KIST Medical College and Teaching Hospital, Imadol, Lalitpur, Nepal

**Keywords:** *diet*, *exercise*, *healthy lifestyles*, *medical students*, *sleep*

## Abstract

The journey of five to six years of medical school is a mixture of different emotions ranging from happiness to stress, anxiety and even mental breakdowns. Diet, sleep and exercise are considered to be three integral components of a healthy life which are often neglected by us, as medical students mostly as a result of such emotional changes in their life. We need a good anount of nutrition to maintain the energy levels for our day-to-day chores, adequate sleep for proper functioning of body and mind and exercise to keep us energized throughout the day. A healthy lifestyle is something that every individual strive towards in their life and as medical students we must always consider about having a healthy lifestyle by balancing our diet, sleep, and exercise.

## INTRODUCTION

The life as a medical student can be hectic at times mostly due to lack of time management for studies, healthy lifestyle, personal life, family time and other different aspects of life. In the meantime, we try to balance out these various aspects of life at the cost of our health. Often we end up having health issues that ultimately hampers all other aspects of our life and get stuck in the vicious cycle of failure to manage time. For a healthy life, diet, sleep, and exercise are all extremely crucial to each other that the whole balance of a healthy life gets disarrayed even if one of them is neglected. Thus, it is a must to balance out our life with a healthy lifestyle as it will eventually influence a patient's health.

## DIETARY HABIT

Healthy students can be better learners and can grasp things with more ease. According to a research, academic achievements are highly influenced by our diet and the way we plan our diet.^1^ Adequate nutrition is an important pillar of good health and as medical students, taking care of our own diet and nutrition should always be of utmost priority. As budding doctors, implementation of "balanced diet" is more important than having knowledge about a healthy diet.

Since the "nutrition" aspect of life mostly gets neglected as medical students, most of them suffer from overweight, obesity or even under-nourished due to overeating of junk food, sedentary lifestyle, ignorance of diet due to exam stress, unsatisfactory hostel food, homesickness, and so on. This directly affects an individual's personality, health, and academic performance.^2^

According to a research conducted among medical students of Nepal, almost 15% were overweight and 12% were underweight.^2^ Therefore, we find that there is a need for coordinated efforts to promote healthy eating habits among medical students to create a healthy tomorrow.

## SLEEP PATTERN

Good quality and adequate amounts of sleep are very important in order to have better cognitive performance, mental health, academic performance and to avoid various health problems. In this global era, we find that poor sleep is very common in individuals and even more among medical students as they carry a huge academic load than others. Other factors may include stress, anxiety, the pressure of maintaining grade points, feeling of inferiority, homesickness, monotonous life, procrastination, lack of organization in tasks, lack of attention, meditation, and so on.

According to a study done among Kathmandu medical students, 96 of the 217 participants (44%) experienced poor sleep, which was more common in women. The mean duration of sleep among medical students was approximately 6.5 hours which was even lesser during exam time.^4^ Thus, a significant number of medical students were found to have a poor quality of sleep which may affect their academic performance and lead them to frustration.

Poor quality of sleep not only leads to deterioration of academic performance and mental health but also may have a long-term impact on their health.^5^ Therefore, efforts must be directed towards educating them about the importance of quality sleep as well as proper time management skills.

## PHYSICAL ACTIVITY

Medical students have substantial knowledge of the benefits of regular physical activity. They can influence their patients' attitudes towards physical activity and can become role models for their patients in the future. But the role of exercise and physical activity is still underestimated by medical students.

There might be several reasons that lead them to compromise toing exercise. Some of the reasons could be due to mis-management of time, hectic study schedules, academic stress, lack of encouragement, motivation, setting unrealistic goals, lack of persistence, seeking immediate results and so on. Several authors have observed a positive relationship between physical fitness and students' academic results.^1^ Regular exercise during young age has also been shown to prevent a person from various chronic illnesses such as diabetes, hypertension, obesity, cardiovascular diseases, etc. during old age.

According to various research, despite being aware of the benefits of exercise, they didn't meet the recommended level of physical activity. In a research conducted on students of health science at Chitwan district, more than 2/3^rd^ of students reported their status as low active. Nearly 7% were physically inactive. A total of 93% met the minimum WHO recommendation for physical activity (>600 MET-minutes/week).^1^ Since there was a huge proportion of students with a low level of physical activity, there is a need for focusing on health information systems regarding the importance of physical activity among medical students.

## SOME ADVICES AS A MEDICAL STUDENT

Thus, diet, sleep, and exercise should all be balanced to improve the mental, as well as physical health and hence increase the efficiency of a medical student.

### DIET

We've all come across this at some point in our lives. When it comes to our productivity, it's actually spot-on. Food has a direct impact on our cognitive performance, which is why a poor decision at lunch can derail an entire day. It is advisable to at least start your day with a healthy breakfast like green salads, fresh fruits and an egg. During lunch and dinner, spicy and fatty food can be avoided. Also hydration is crucial so regular intake of plain drinking water should be maintained. Also, it is better to avoid junk foods and consume healthy food items only as much as possible.

### SLEEP

Maintaining a proper sleep hygiene and sticking to it is important. For this, reduction of the screen time, particularly at the evening, avoidance of alcoholic beverages and coffee during evening, proper maintainance of a clean fresh bedroom are advised and can help medical students to get a good night's sleep. Further, if one has difficulty in going to the bed on time and waking up early, modalities such as setting reminders and alarms can help.

### EXERCISE

Daily exercise for at least 30 minutes is recommended as it not only rejuvenates the body but also decreases stress, boosts the immune system, keeps you productive, and helps you sleep better at night. If one cannot allocate separate hours to head to the gym, exercise at the comfort of your house can also be done ensuring adequate time at your own possible place. Exercise in any forms such as at the gym, yoga, swimming or walks are advised.

## WAY FORWARD

We, as medical students must always try to maintain the three important pillars of balanced healthy life i.e. healthy diet, quality sleep, and regular exercise to be able to function properly. Although medical students have adequate knowledge about how to maintain a healthy life, we are still lagging behind to put it into practice. Therefore, we should always work hand-in-hand and encourage our friends and colleagues to focus on these three important aspects of our health for attaining a better life.
